# A Straightforward Methodology for the Quantification of Long Chain Branches in Polyethylene by ^13^C NMR Spectroscopy

**DOI:** 10.3390/polym17091274

**Published:** 2025-05-07

**Authors:** Francesco Zaccaria, Andrea Pucciarelli, Roberta Cipullo, Vincenzo Busico

**Affiliations:** 1 Department of Chemical Science, Federico II University of Naples, 80126 Naples, Italybusico@unina.it (V.B.); 2 Scuola Superiore Meridionale, Largo San Marcellino, 80138 Naples, Italy; a.pucciarelli@ssmeridionale.it

**Keywords:** polyethylene, long chain branches, microstructure, NMR spectroscopy

## Abstract

Formation of long chain branches (LCB) in polyethylene (PE), via incorporation of in situ generated vinyl macromonomers, is known to affect material properties dramatically, making their detection and quantification of primary importance. ^13^C NMR spectroscopy is the archetypal technique for the analysis of polymer microstructure, yet it suffers from major limitations in the analysis of LCB in polyethylene, primarily in terms of resolution. Herein, we propose a simple and effective methodology for detecting and quantifying LCB based on the analysis of C atoms in β-position with respect to the branching point. By analyzing model ethylene/α-olefin copolymers bearing methyl, ethyl, butyl, hexyl or tetradecyl chain branches, we show how the C_β_ resonances can be used to discriminate between shorter or longer branches. Importantly, the proposed method allows the most critical discrimination between hexyl-type branches and LCB, with an up to three-fold detection enhancement with respect to previously proposed procedures based on the analysis of the methine carbons. The proposed approach is then tested on a representative industrial sample of HDPE, proving that it is suitable to detect very small amounts of LCB.

## 1. Introduction

One of the main reasons behind the “success story” [1] of polyolefins lies in the possibility of widely tuning material properties by tailoring polymer microstructure [1,2,3,4]. Polyethylene (PE), the most widely used thermoplastic material, represents a prominent example in this respect. The possibility of accessing a large variety of PE grades makes this material suitable for a broad range of applications, from packaging to construction materials, fibers and more [1,2]. The three main classes of PE consist of low density (LDPE), linear low density (LLDPE) and high density (HDPE) polyethylene, which differ primarily for the type and degree of branching of the polymer chain. LDPE is produced via radical processes that provide low crystallinity, hyperbranched polymers. LLDPE and HDPE are instead essentially linear polymers produced via catalytic ethylene/α-olefin copolymerization or ethene homopolymerization, respectively [5,6].

**Figure 1:**
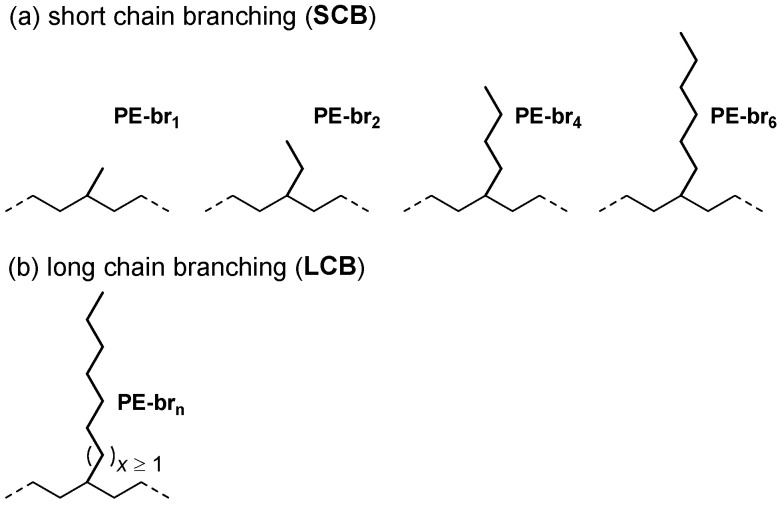
Representative (**a**) short and (**b**) long chain branches in polyethylene.

The typical α-olefins used in the synthesis of LLDPE are 1-butene, 1-hexene and 1-octene, which introduce short chain branches (SCB; Figure 1) consisting of two (PE-br_2_), four (PE-br_4_) and six (PE-br_6_) carbon atoms in the otherwise perfectly linear polyethylene backbone, respectively [7]. The amount, distribution and length of these SCB critically influence the macroscopic properties of the final material [8,9,10,11,12]. SCB of only one carbon can also be introduced in PE via copolymerization of ethylene and propene, which however does not provide a thermoplastic material but the so-called ethylene-propylene rubber (EPR; Figure 1) [4,13].

Importantly, chain branches other than those expected based on the co-monomers used for the synthesis are often found in LLDPE and even HDPE. The most important ones are the so-called long chain branches (LCB; Figure 1). These longer ramifications derive from incorporation of vinyl-terminated macromonomers that are produced in situ during polymerization, especially when the process is conducted in the absence of hydrogen [14]. Although SCB can also form via similar processes [15,16], detecting LCB in PE is of primary importance [17] since they dramatically affect the final physical and rheological properties of the material [18,19].

The presence of LCB can be inferred from the analysis of polymer properties, for instance by rheology [20,21,22] or triple-detector gel permeation chromatography [23]. Nevertheless, direct spectroscopic detection and quantification of LCB content is generally preferable. IR spectroscopy is convenient in terms of costs and analysis time [24] but suffers from major complications due to interferences and dependence of absorbance frequency and absorptivity on the type of sample, which strongly limits applications in this field [17]. Alternatively, ^13^C NMR spectroscopy can provide truly quantitative and absolute quantification of LCB [25], even though some limitations also apply to this technique [26,27].

The typical way to detect ramifications in PE by ^13^C NMR is the analysis of the branch carbons, that is, the methine groups shared between the polymer backbone and the branch [26]. However, this approach has two main drawbacks. The first relates to the characteristic low sensitivity of ^13^C NMR spectroscopy, which is critical especially for the detection of small amounts of microstructural fragments: modern spectrometers allow circumventing this limitation not only by increasing the number of transients (and therefore acquisition time) but also utilizing high temperature cryoprobes [28,29,30,31] and/or adopting ad hoc developed pulse sequences [16,32,33,34,35].

The second limitation is instead more problematic and relates to the resolution needed to distinguish different types of branches. In fact, SCB containing up to five carbon atoms can be easily discriminated against each other, while the branch carbon of all side chains having a number of carbon atoms ≥ 6 resonate practically at the same chemical shift under typical experimental conditions (i.e., in tetrachloroethane-*d*_4_ at 393 K). This implies that LCB cannot be distinguished from hexyl-type SCB, like those deriving from 1-octene incorporation, based solely on the analysis of methine resonances.

Several approaches have been proposed to address this problem, such as working in different and more exotic solvents (e.g., 1-chloronaphtalene/*p*-dichlorobenzene-*d*_4_ 9:1 *v*/*v* mixture) to improve peak separation [36,37]. Additionally, Zhou and coworkers have recently proposed advanced INADEQUATE-type pulse sequences to further improve resolution and sensitivity of NMR spectra [32,38]. Although these strategies have proved highly effective, they can hardly be applied on a routine basis for LLDPE and HDPE characterization, due to the costs of solvent mixtures and the pitfalls behind the reliable execution of complex NMR experiments [32,38]. Simplicity and speed are becoming increasingly important in the characterization of polyolefins [39,40,41,42,43].

NMR detection of side chains in PE can also be achieved by analyzing diagnostic nuclei other than the methine carbon [16,33,34,44,45,46,47,48,49]. The C atoms in α-position to the methine group are the most widely investigated owing to their proximity to the branching, but resolution remains problematic in this case [26,46,47,48]. Conversely, to the best of our knowledge, the methylene C atoms in β-position have received practically no attention for the quantification of SCB and LCB. On the one hand, the longer distance of C_β_ from the branching point makes these nuclei seemingly less diagnostic compared to the methine and C_α_. On the other hand, while still being in the proximity of the branching point, the C_β_ of the branch itself is closer than the methine and C_α_ to the tail of the side chain and—consequently—it is more sensitive to its length: it therefore represents a potentially better probe to discriminate hexyl-type SCB from LCB. Herein, we explore this approach and propose a straightforward methodology based on the analysis of C_β_ resonances for the detection and quantification of LCB in PE. This methodology requires typical 1D ^13^C NMR spectra and it is therefore easily accessible to any laboratory equipped for the NMR characterization of polyolefin.

## 2. Materials and Methods

Model ethylene/α-olefin copolymers were synthesized with typical metallocene catalysts using a Freeslate PPR platform according to well established procedures [50,51,52]. Prior to the execution of a polymerization library, the PPR modules undergo ‘bake-and-purge’ cycles overnight (4 h at 85 °C with intermittent dry N_2_ flow), to remove any contaminants and left-overs from previous experiments. After cooling to glovebox temperature, the module stir tops are taken off, and the 48 cells are fitted with disposable 10 mL glass inserts (pre-weighed in a Mettler-Toledo Bohdan Balance Automator) and polyether ether ketone (PEEK) stir paddles. The stir tops are then set back in place, and N_2_ in the reactors is replaced with ethene (ambient pressure). The cells are then loaded with the appropriate amounts of toluene and co-monomer. The system is then thermostated at the reaction temperature and brought to 65 psi of pressure with ethene. At this point, the catalyst injection sequence is started; aliquots of (a) a toluene ‘chaser’, (b) a toluene solution of catalyst, (c) a toluene spacer, (d) a toluene solution of the proper activator and (e) a toluene ‘buffer’, all separated by nitrogen gaps, are uploaded into the needle and subsequently injected into the cell of destination in reverse order, thus starting the reaction. This is left to proceed under stirring (800 rpm) at constant temperature and pressure with feed of ethene on demand for 10 min and quenched by over-pressurizing the cell with 50 psi (3.4 bar) of dry air (preferred over other possible catalyst quenchers because in case of cell or quench line leakage oxygen is promptly detected by the dedicated glove-box sensor). Conversion of co-monomer was kept below 15% to ensure a constant co-monomer concentration. Once all cells have been quenched, the modules are cooled down to glovebox temperature and vented, the stir-tops are removed, and the glass inserts containing the reaction phases are taken out and transferred to a centrifugal evaporator (Genevac EZ-2 Plus or Martin Christ RVC 2-33 CDplus), where all volatiles are removed, and the polymers are thoroughly dried overnight. Reaction yields are double-checked against online monomer conversion measurements by robotically weighing the dry polymers while still in the reaction vials, subtracting the pre-recorded tare. Polymer aliquots are then sent to the characterizations.

The industrial HDPE sample was kindly provided by SABIC Europe (Geleen, The Netherlands).

NMR spectra were recorded following established procedures [3,31,42] at 393 K using a Bruker Avance III 400 spectrometer equipped with a high-temperature cryoprobe for 5 mm OD tubes, on ~45 mg mL^−1^ polymer solutions in tetrachloroethane-*d*_2_ (with BHT added as a stabilizer, [BHT] = 0.4 mg mL^−1^). Quantitative ^13^C NMR spectra were recorded using a 45° pulse sequence with a modified WALTZ16 sequence (BI_WALTZ16_32 by Bruker) for broad-band proton decoupling (acquisition time, 1.4 s; time domain, 64 k; relaxation delay, 5.0 s; 1–15 K transients).

## 3. Results and Discussion

### 3.1. Detection of LCB in the Absence of Overlapping SCB

To provide an overview of the most common ethylene/α-olefin copolymers, five representative samples of PE containing methyl (PE-br_1_), ethyl (PE-br_2_), butyl (PE-br_4_), hexyl (PE-br_6_) or tetradecyl (PE-br_14_, Figure 1) side chains were characterized by ^13^C NMR spectroscopy (Figure 1 and Figures S1–S5). Their NMR spectra were acquired with a medium-high field (400 MHz for ^1^H) spectrometer equipped with a 5 mm high temperature cryoprobe under typical experimental conditions (see Experimental Part for details). Satisfactory signal-to-noise ratios could be obtained within ~0.5 h acquisition time, which can be considered short in this context and compatible with relatively fast analytics of polyolefins [42].

**Figure 2:**
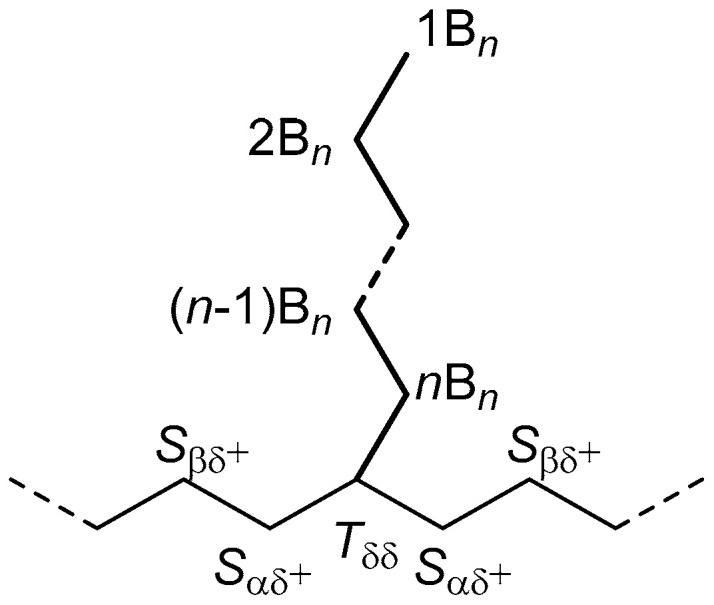
Nomenclature of relevant ^13^C NMR resonances.

NMR signals were assigned according to the literature [53,54,55], using the nomenclature proposed by Randall [26]. The carbon atoms of the side chains are indicated as *B_n_*, where *n* is the total number of C atoms of the ramification, preceded by a number indicating the position of each carbon (with the terminal methyl corresponding to position 1; Figure 2). In the polyethylene backbone, the branch carbon is indicated as *T*_δδ_, while the C atoms in α and β position with respect to this methine group are named *S*_αδ_^+^ and *S*_βδ_^+^, respectively (Figure 2). 

**Figure 3:**
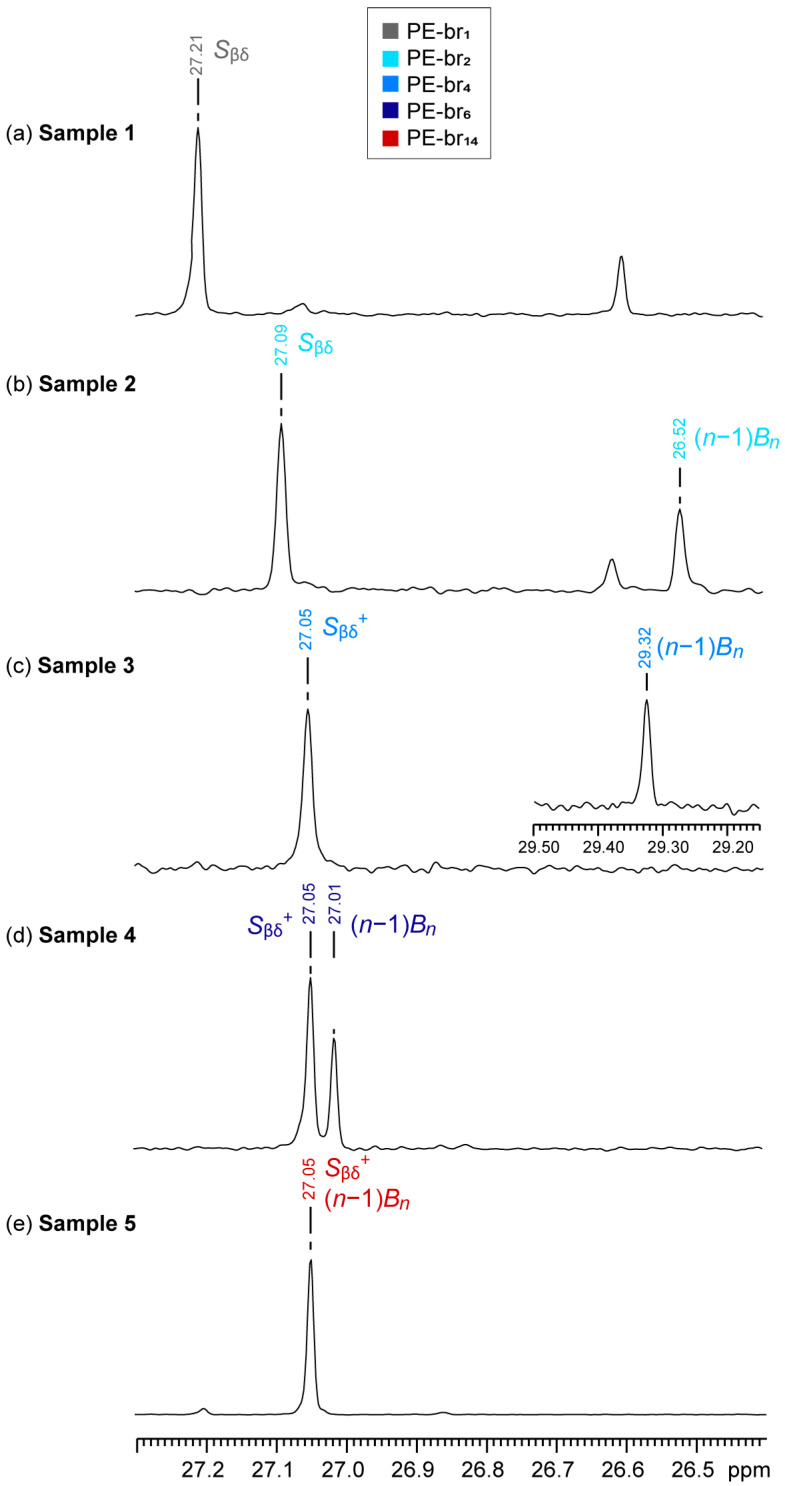
Selected regions of ^13^C NMR spectra (tetrachloroethane-*d*_4_, 393 K) of the five representative PE samples studied containing (**a**) methyl (PE-br_1_), (**b**) ethyl (PE-br_2_), (**c**) butyl (PE-br_4_), (**d**) hexyl (PE-br_6_) or (**e**) tetradecyl (PE-br_14_) side chains.

Figure 3 shows the ^13^C NMR spectra of the five PE samples, expanded in the C_β_ region. PE-br_1_ only has *S*_βδ_^+^ carbons, which resonate at a characteristic δ(^13^C) = 27.21 ppm (Figure 3a). As expected, it can be easily distinguished from PE-br_2_ and PE-br_4_ owing to their distinctively different *S*_βδ_^+^ signals resonating at 27.08 vs. 27.05 ppm, respectively (Figure 3b,c). Furthermore, PE-br_2_ is particularly easy to recognize since the 2*B_n_* carbon corresponds to a methyl group appearing at δ(^13^C) = 26.52 ppm (Figure 3b). Discriminating between PE-br_4_ and PE-br_6_ is less straightforward but still possible, since *S*_βδ_^+^ chemical shifts are identical (27.05 ppm), while (*n* − 1)*B_n_* differ significantly (29.32 vs. 27.01 ppm; Figure 3c,d).

The problem is more complex when comparing longer ramifications, namely PE-br_6_ and PE-br_14_. For side chains with *n* ≥ 6 carbon atoms, the C_β_ of the branch become indistinguishable from those of the main chain, implying that the signals of (*n* − 1)*B_n_* overlap with that of *S*_βδ_^+^ for PE-br_14_ and they both appear at the same chemical shift of *S*_βδ_^+^ in PE-br_6_ (27.05 ppm; Figure 3d,e). Several scenarios can be envisaged to distinguish these two types of branches. The simplest one is exemplified by Sample 5 in Figure 3d, where the absence of the (*n* − 1)*B_n_* resonance at 27.01 ppm indicates that no PE-br_6_ is present and, consequently, the peak at 27.05 ppm can be assigned totally to the three equivalent C_β_ of PE-br_14_.

### 3.2. Detection of LCB in the Presence of Overlapping SCB

**Figure 4:**
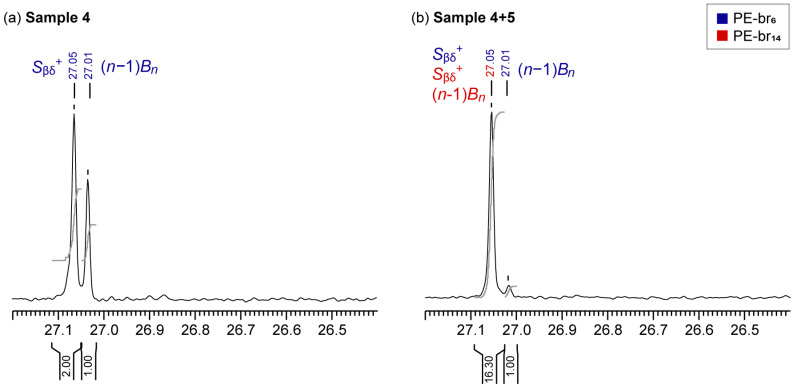
Selected regions of ^13^C NMR spectra (tetrachloroethane-*d*_4_, 393 K) of (**a**) the Sample 4, containing only hexyl-type SCB, and (**b**) a mixture of Sample 4 and 5, containing also LCB. Grey curves are integral curves.

In cases where both signals at 27.05 and 27.01 ppm are present, PE-br_6_ and PE-br_14_ cannot be distinguished solely based on the number and type of signals and a more detailed quantitative analysis is required. Figure 4a shows the ^13^C NMR spectrum of the above discussed ethylene/1-octene copolymer Sample 4. The 2:1 integral ratio between the signals at 27.05 and 27.01 ppm is exactly that expected between the two *S*_βδ_^+^ and the one (*n* − 1)*B_n_* carbons of PE-br_6_, therefore indicating that only hexyl type SCB are present in this case.

Figure 4b shows instead the spectrum of a mixture of Sample 4, bearing PE-br_6_ branches, and Sample 5, bearing PE-br_14_. The same two peaks are present at 27.05 and 27.01 ppm, yet their integral ratio deviates from the expected 2:1 for hexyl-type SCB alone. This indicates that LCB are present as well, as they contribute to the area of the sole peak at 27.05 ppm. In this case, the contribution of LCB (*I*_Cβ, LCB_) to the C_β_ resonance (*I*_27.05 ppm_) can be determined by subtracting that of PE-br_6_, estimated from the peak at 27.01 ppm (*I*_27.01 ppm_), according to the following equation:

(1) $$I_{C_{\beta },\;LCB}=\frac{I_{27.50\;ppm}-2\times I_{27.01\;ppm}}{3}$$

which accounts for the presence of three magnetically equivalent C_β_ for LCB and of two magnetically equivalent *S*_βδ_^+^ in PE-br_6_. Importantly, the same strategy cannot be applied to other nuclei such as C_α_, since *S*_αδ_^+^ overlaps with *n*B_n_ for both PE-br_6_ and LCB and, consequently, there is no way to discriminate the individual contributions when both fragments are present [26,46,47,48].

It is worth emphasizing that, albeit being easily recognizable based on diagnostic resonances other than C_β_, PE-br_4_ also contributes to the peak at 27.50 ppm (Figure 3c), and it should be therefore considered in the estimation of LCB. To this aim, Equation (1) can be generalized to account for all the most common SCB as follows:

(2) $$I_{C_{\beta },\;LCB}=\frac{I_{27.50\;ppm}-2\times I_{27.01\;ppm}-2\times I_{29.32\;ppm}}{3}$$

where integral of the peak of the (*n* − 1)*B_n_* carbon at 29.32 ppm is used to estimate the contribution of PE-br_4_ to the peak at 27.50 ppm.

The C_β_ appear, therefore, to provide a good compromise between the diagnostic nature of their NMR signals and the resolution of SCB vs. LCB resonances. It is fair to admit that when the amount of LCB is very low with respect to that of PE-br_6_, LCB’s contribution to *I*_27.05ppm_ might be in the same order of experimental uncertainty of NMR integrals. In these cases, the use of 10 mm HT cryoprobes coupled with (very) long acquisition times is mandatory. It should be noted, however, that the presence of three equivalent C_β_ in LCB offers a 3-fold sensitivity gain compared to one-carbon *T*_δδ_ signals, representing a strong advantage in terms of sensitivity of the method proposed therein compared to those based on the analysis of methine resonances.

### 3.3. Analysis of a Representative Industrial Sample

As an exemplifying application of the proposed approach, we analyzed a representative industrial HDPE sample. Previous rheological characterization indicated the presence of small amounts of LCB, on the order of 1 LCB/10,000 C.

**Figure 5:**
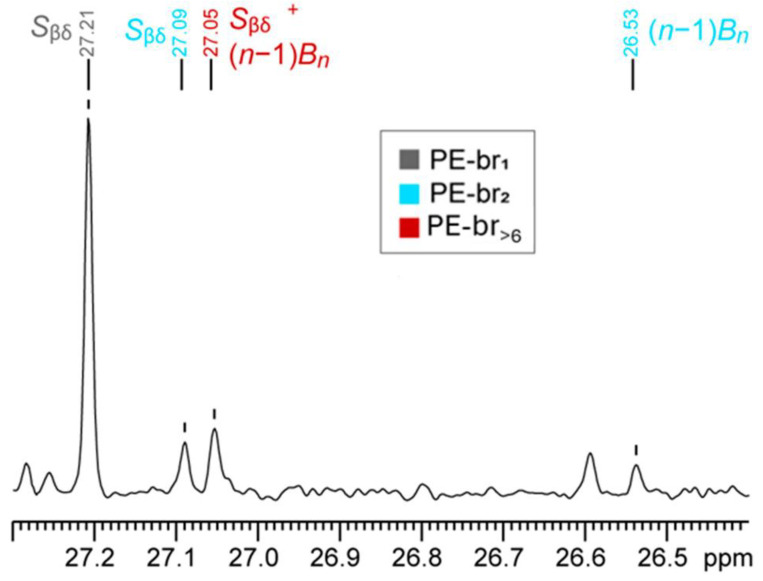
Selected region of ^13^C NMR spectrum (tetrachloroethane-*d*_4_, 393 K) of an industrial HDPE sample.

The ^13^C NMR spectrum of this HDPE sample shows several signals in the C_β_ region (Figure 5), indicating the presence of various chain branches even though no co-monomer was used in the synthesis. The peak at δ(^13^C) = 27.21 ppm can be easily assigned to methyl branches (cf. Figure 3a). Likewise, the peaks at 27.09 and 26.53 are diagnostic of the presence of PE-br_2_ (cf. Figure 3b).

Importantly, a peak at 27.05 ppm is clearly visible. The absence of a resonance at 27.02 ppm, corresponding to *S*_βδ_^+^ of PE-br_6_, and of a signal at 29.32 ppm, corresponding to *S*_βδ_^+^ of PE-br_4_, implies that the resonance at 27.05 ppm is only due to the three equivalent C_β_ of LCB. By peak integration, it is possible to quantitatively determine the amount of LCB [26]. The calculated value of 0.7 LCB/10,000 C is nicely in line with rheological measurements, demonstrating the effectiveness of the proposed approach in detecting accurately even tiny amounts of such relevant fragments, down to at least 0.2–0.3 LCB/10,000 C.

## 4. Conclusions

Detection and quantification of LCB in PE resins is of crucial importance for understanding macroscopic properties of this widely used material. Herein, we propose a straightforward approach to the evaluation of this key feature, based on the careful analysis of ^13^C NMR signals of carbon nuclei in β-position with respect to the branching point. This methodology requires simple 1D NMR spectra acquired under typical conditions for polyolefins, i.e., in tetrachloroethane-*d*_4_ at 393 K, and allows effective discrimination between SCB and LCB. In particular, the critical discrimination between hexyl-type SCB and LCB can be achieved by analyzing C resonances at 27.05 and 27.01 ppm as follows:

1. If only one C_β_ signal at 27.05 ppm is present, the polymer contains only LCB
2. If two C_β_ signals at 27.05 and 27.01 ppm are present and exhibit a 2:1 integral ratio, the polymer contains only hexyl-type SCB
3. If two C_β_ signals at 27.05 and 27.01 ppm are present and exhibit an integral ratio higher than 2:1, the polymer contains both hexyl-type SCB and LCB, and the contribution of the latter can be estimated using Equation (1) (or Equation (2)).

This approach offers important advantages in terms of simplicity and sensitivity compared with established methods based on the analysis of *T*_δδ_, since it does not require sophisticated solvent mixtures nor pulse sequences and—more importantly—warrants a 3-fold sensitivity increase due to the presence of three equivalent C_β_ in LCB. The effectiveness of this approach was demonstrated in the analysis of an industrial HDPE sample containing 0.7 LCB/10,000 C, in line with rheological data. Considering that modern spectrometers—ideally equipped with 10 mm cryoprobes—allow acquiring ^13^C NMR spectra with very high signal-to-noise ratio in tens of minutes, the analysis of LCB proposed herein represents an important advancement towards accurate and information-rich characterization of polyolefins.

## Data Availability

The original contributions presented in this study are included in the article/Supplementary Materials. Further inquiries can be directed to the corresponding author.

## Supplementary Materials

The following supporting information can be downloaded at: https://www.mdpi.com/article/10.3390/polym17091274/s1, Figure S1: ^13^C NMR spectrum of Sample 1; Figure S2: ^13^C NMR spectrum of Sample 2; Figure S3: ^13^C NMR spectrum of Sample 3; Figure S4: ^13^C NMR spectrum of Sample 4; Figure S5: ^13^C NMR spectrum of Sample 5; Figure S6: ^13^C NMR spectrum of the industrial HDPE sample.

## Abbreviations

The following abbreviations are used in this manuscript:

PE	Polyethylene
LDPE	Low-density polyethylene
LLDPE	Linear low-density polyethylene
HDPE	High-density polyethylene
LCB	Long chain branches
SCB	Short chain branches
