# Supplementary material for: A Straightforward Methodology for the Quantification of Long Chain Branches in Polyethylene by 13C NMR Spectroscopy
Source: Polymers (Basel). 2025 May 7;17(9):1274. doi: 10.3390/polym17091274 (PMC12074469; doi:10.3390/polym17091274)
Supplement: Supplementary file 1 [file polymers-17-01274-s001.zip › polymers-3615877-supplementary.pdf]

Supporting Information

A straightforward methodology for the quantification of long chain branches in polyethylene by ^{13}C NMR spectroscopy

Francesco Zaccaria [1], Andrea Pucciarelli [2], Roberta Cipullo [1,*], and Vincenzo Busico [1]

1 Department of Chemical Science, Federico II University of Naples, Naples, Italy
2 Scuola Superiore Meridionale, Largo San Marcellino, Naples, Italy
Correspondence: rcipullo@unina.it

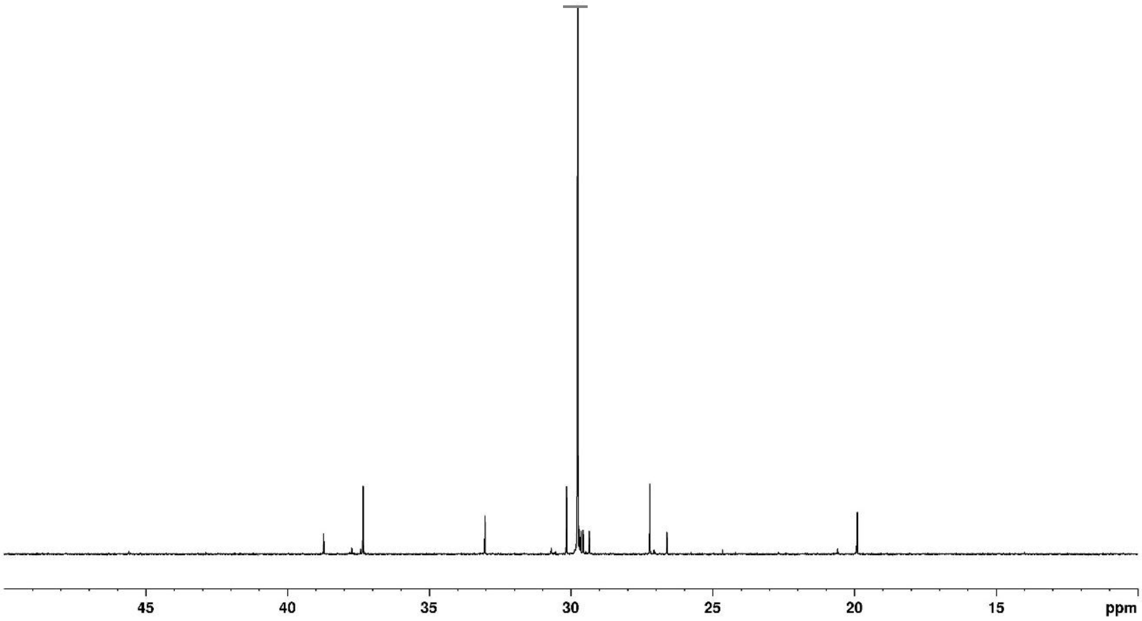

Figure S1. ^{13}C NMR spectrum of **Sample 1**.

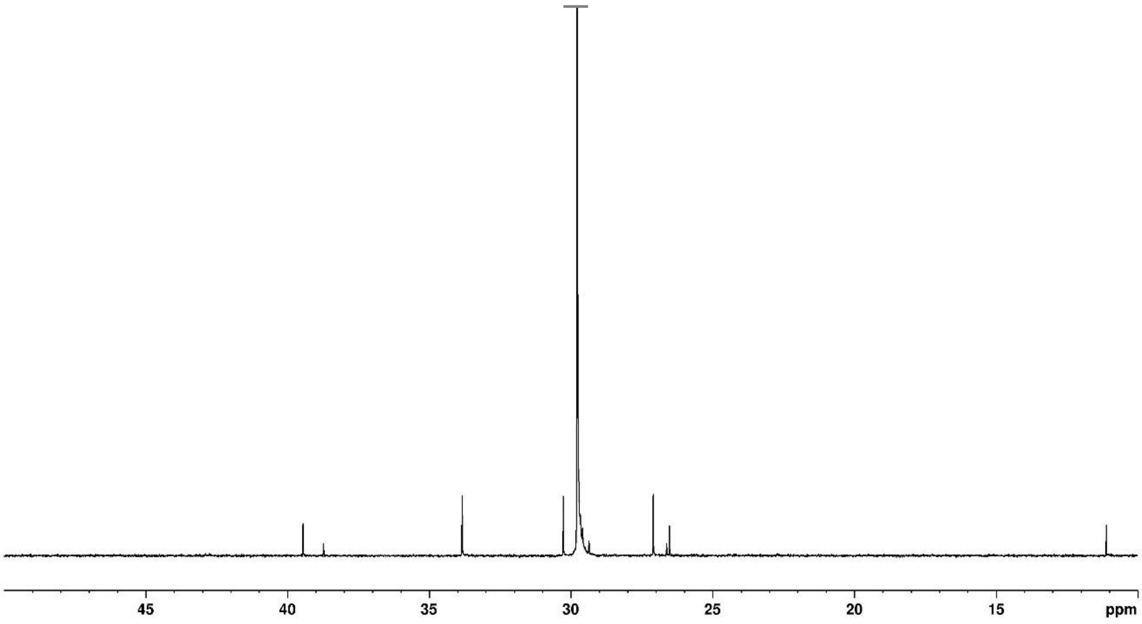

Figure S2. ^{13}C NMR spectrum of **Sample 2**.

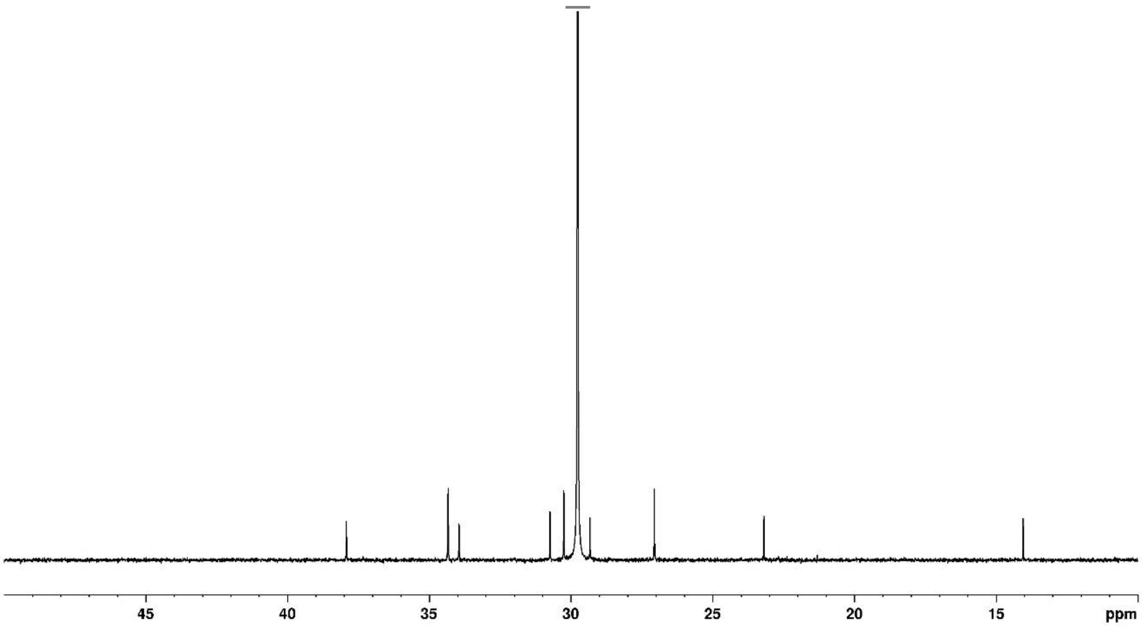

Figure S3. ^{13}C NMR spectrum of **Sample 3**.

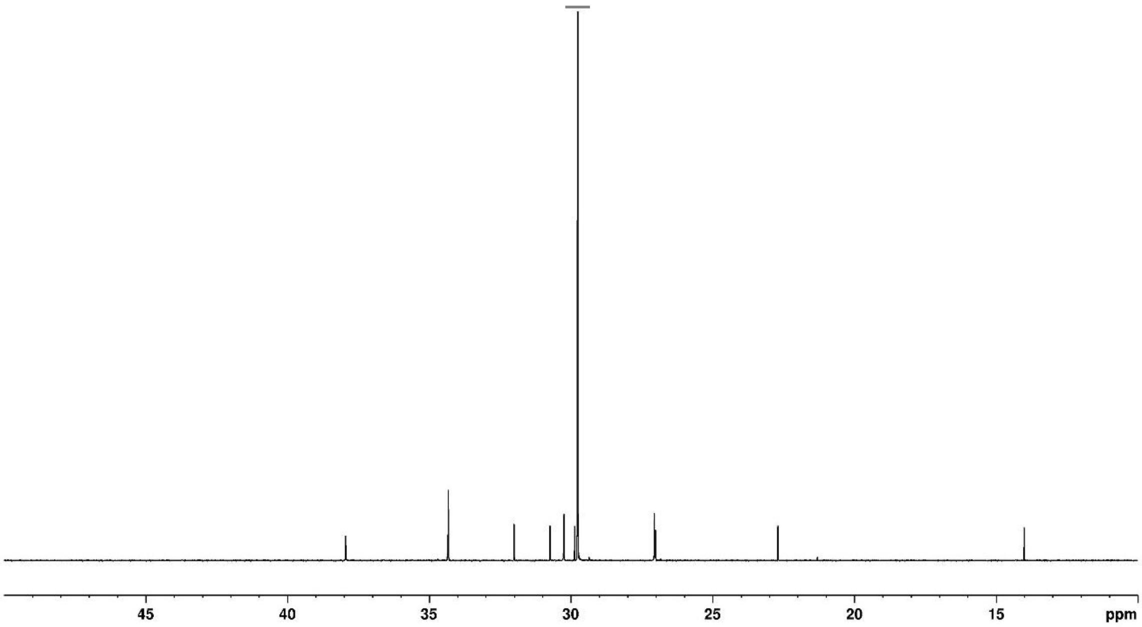

Figure S4. ^{13}C NMR spectrum of **Sample 4**.

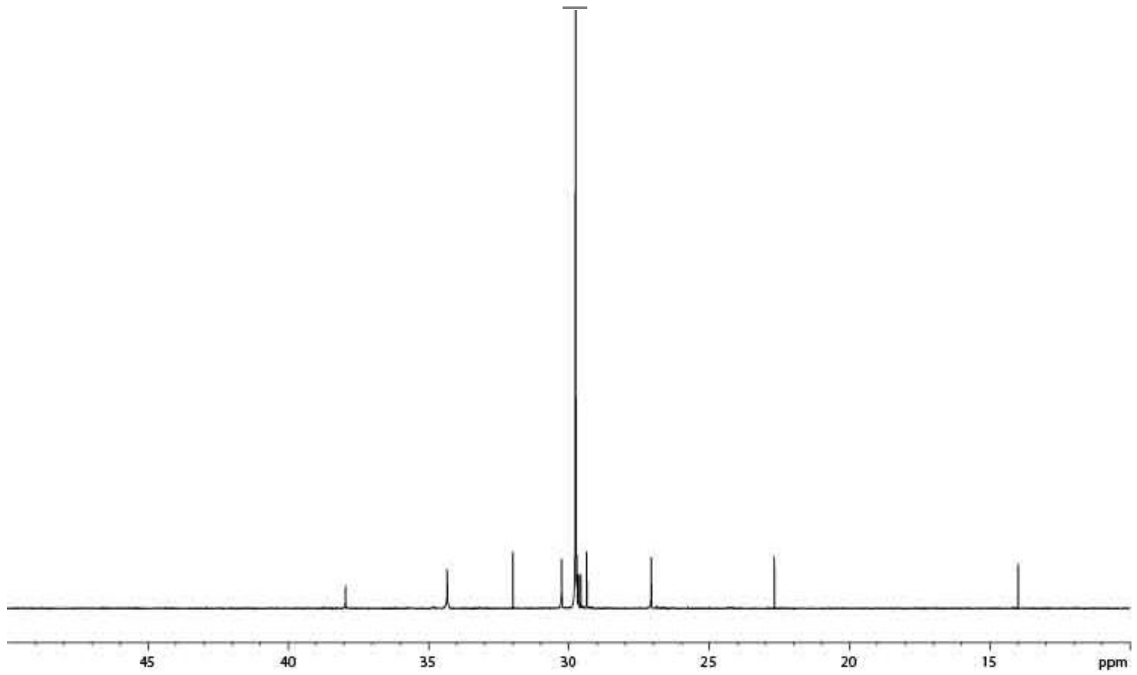

Figure S5. ^{13}C NMR spectrum of **Sample 5**.

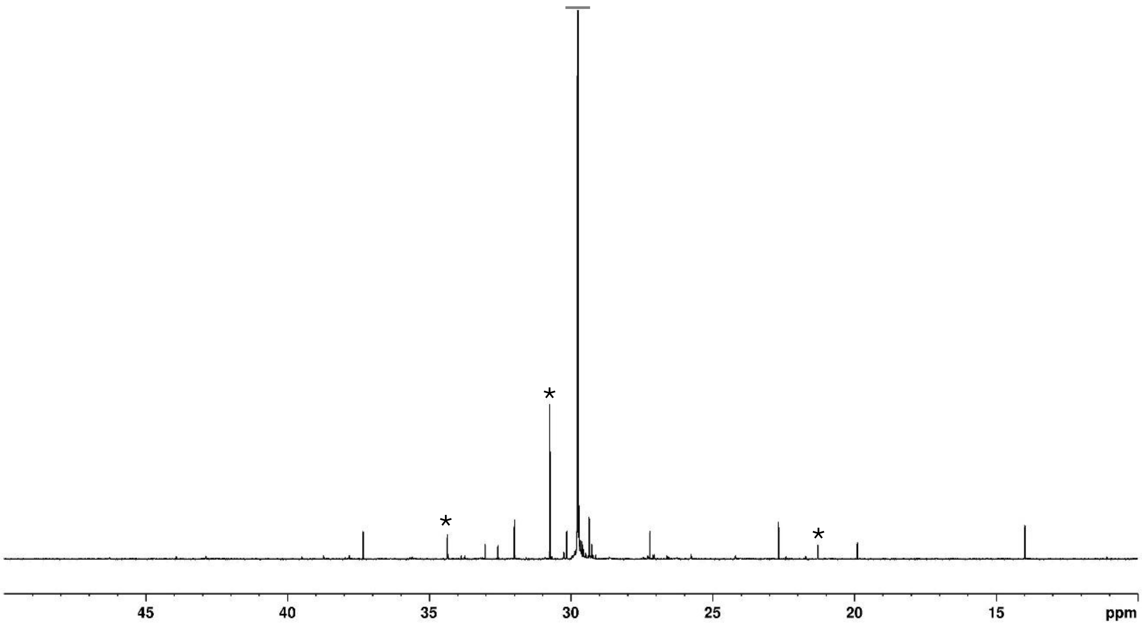

Figure S6. ^{13}C NMR spectrum of the industrial HDPE sample. * = signals of BHT used as stabilizer.
